# Reprogramming the Methylome: Erasing Memory and Creating Diversity

**DOI:** 10.1016/j.stem.2014.05.008

**Published:** 2014-06-05

**Authors:** Heather J. Lee, Timothy A. Hore, Wolf Reik

**Affiliations:** 1 Epigenetics Programme, The Babraham Institute, Cambridge, CB22 3AT, UK; 2 Wellcome Trust Sanger Institute, Hinxton CB10 1SA, UK; 3 Centre for Trophoblast Research, University of Cambridge, Cambridge CB2 3EG, UK

## Abstract

The inheritance of epigenetic marks, in particular DNA methylation, provides a molecular memory that ensures faithful commitment to transcriptional programs during mammalian development. Epigenetic reprogramming results in global hypomethylation of the genome together with a profound loss of memory, which underlies naive pluripotency. Such global reprogramming occurs in primordial germ cells, early embryos, and embryonic stem cells where reciprocal molecular links connect the methylation machinery to pluripotency. Priming for differentiation is initiated upon exit from pluripotency, and we propose that epigenetic mechanisms create diversity of transcriptional states, which help with symmetry breaking during cell fate decisions and lineage commitment.

## Teaser

This Perspective discusses synergistic mechanisms that result in epigenetic memory loss in PGCs, the early embryo, and naive ESCs. Links between pluripotency and loss of epigenetic memory and connections between heterogeneous DNA methylation and lineage priming are also explored.

## Main Text

### Introduction

Cellular identity is maintained by epigenetic memory, which in part reflects the developmental history of a cell (Reik, 2007; Sasaki and Matsui, 2008; Surani et al., 2007). Epigenetic memory relies upon the faithful inheritance of epigenetic marks (such as DNA methylation or histone modifications) during cell division. This heritability is a hallmark of epigenetic regulation (Russo et al., 1996) and, in the case of DNA methylation, is brought about by a well-understood mechanism.

In mammals, cytosine methylation occurs mostly in the context of palindromic CpG dinucleotides, where methylation occurs on both DNA strands in an antiparallel fashion. During DNA replication, the CpG methylation pattern can be copied from the template strand to the newly synthesized DNA strand. The UHRF1 protein recognizes hemimethylated sites and recruits the maintenance DNA methyltransferase, DNMT1, to methylate the newly synthesized strand. In contrast, the de novo methyltransferases DNMT3A/B, and their cofactor DNMT3L, do not depend on a hemimethylated template and can establish new patterns of DNA methylation. As well as methylating CpG dinucleotides, these enzymes are thought to be responsible for the low, but consistent, levels of non-CpG methylation observed in oocytes, prospermatogonia, embryonic stem cells (ESCs), and neural cells (Hon et al., 2013; Lister et al., 2009; Shirane et al., 2013). There is no apparent epigenetic memory of non-CpG methylation, as there is no mechanism to maintain these marks following replication.

**Figure 1:**
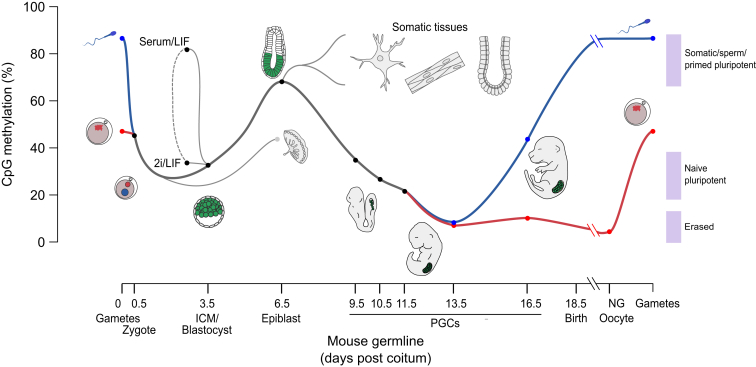
Global CpG Methylation Levels in the Mouse Germline, Somatic Tissues, and ESCs The mouse germline undergoes two major waves of demethylation, the first in the early embryo where the paternal genome (blue) is actively demethylated prior to and during replication. Both the paternal and maternal (red) genomes passively lose methylation after this until the blastocyst stage (E3.5). The second wave of demethylation occurs in the primordial germ cells between E6.5 and E13.5 as they emerge from the epiblast. Methylation is then re-established in a sex-specific manner after E13.5 and the nongrowing (NG) oocyte stage, in males and females, respectively, eventually giving rise to mature gametic patterns. Naive and primed ESCs can be cultured from the ICM or be interchanged with each other (dashed line), by growth in either serum or 2i media, respectively. Only naive ESCs display low methylation (∼30%) that corresponds to in vivo pluripotent tissues (shaded boxes on the far right). Erased cells display less than 10% methylation, whereas somatic tissues (derived from the E6.5 epiblast) show consistently high methylation around 70%–85%. The placenta is relatively demethylated compared to somatic tissues and is derived from the blastocyst trophectoderm (E3.5). In order to compare between genome-wide (Ficz et al., 2013; Hon et al., 2013; Kobayashi et al., 2012; Seisenberger et al., 2012; Shirane et al., 2013) and reduced representation bisulfite sequencing data sets (Smallwood et al., 2011; Smith et al., 2012), 100 kb probes not overlapping CpG islands were analyzed as previously (Ficz et al., 2013).

Epigenetic memory is thought to be robust in somatic tissues, where levels of CpG methylation are consistently high (70%–85%) (Hon et al., 2013; Ziller et al., 2013) (Figure 1). Methylation is mainly located in transposons, gene bodies, and intergenic regions, while regions of high CpG density (known as CpG islands, CGIs), often located at gene promoters, are generally kept free of methylation. A minority of CGIs (approximately 10% of a total 23,000 in mouse) are differentially methylated between tissues. In addition, distal regulatory elements, such as enhancers (defined by DNase I hypersensitivity, transcription factor binding, and chromatin modifications), show differential methylation across tissues (Hon et al., 2013; Ziller et al., 2013). Thus, tissues and developmental lineages can be robustly delineated by the extent and pattern of DNA methylation at relatively few genic and nongenic CGIs and enhancers. This epigenetic patterning probably provides a framework for the stability of the 10,000–13,000 genes that are differentially expressed between somatic tissues (Ramsköld et al., 2009). These patterns first arise during early postimplantation development and are dependent on the de novo methyltransferases DNMT3A and B, but the precise mechanisms by which they are generated are not known (Borgel et al., 2010; Smallwood et al., 2011).

In marked contrast to somatic tissues, mammalian primordial germ cells (PGCs), early embryos, and naive embryonic stem cells (ESCs) have methylation levels between 5% and 30% (Figure 1), meaning that in the extreme case they have lost up to 15 million methylated CpGs per haploid genome. Since these hypomethylated cell types are pluripotent, or in the case of PGCs can give rise to cells with pluripotent ability (that is, the capability to differentiate into all cell types of the embryo), it raises the question of whether and how epigenetic reprogramming is connected with developmental capacity.

Here we compare the synergistic mechanisms that result in epigenetic memory loss in PGCs, the early embryo, and naive ESCs, and we explore the link between pluripotency and loss of epigenetic memory. Importantly, as cells exit pluripotency and begin cell fate commitment, they re-establish epigenetic marks. We propose a model whereby heterogeneous patterns of DNA methylation are generated at this time to allow cell lineage priming prior to commitment.

### Synergistic Mechanisms of Epigenetic Reprogramming

#### Primordial Germ Cells

Primordial germ cell (PGC) specification results in the single largest change of DNA methylation in the mammalian life cycle. PGCs differentiate from precursor cells in the epiblast, which at E6.5 is as highly methylated as somatic cells, and over the following 7 days or so lose 90% of their global methylation (Figure 1). Significantly, CGI methylation patterns in early PGCs correlate highly with the epiblast and other somatic tissues but not with the oocyte, the ICM, or any other cells of the preimplantation embryo (Ficz et al., 2013; Seisenberger et al., 2012). Although quantitative single-cell analysis will undoubtedly further illuminate this phenomenon, the presence of an epiblast-derived methylation signature in PGCs almost certainly demonstrates that they are reprogrammed from differentiated tissues rather than being rare remnants of the ICM that have escaped de novo methylation.

**Figure 2:**
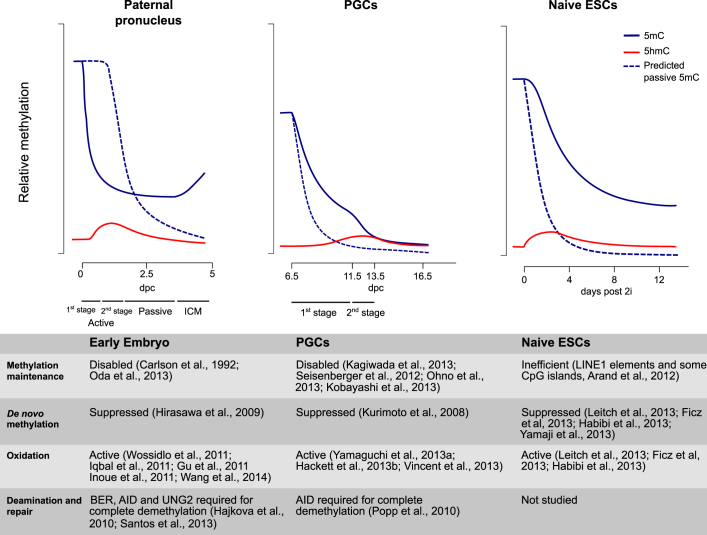
Kinetics of Genome-wide Demethylation in Early Embryos, PGCs, and ESCs Impairment of methylation establishment and maintenance contributes to genome-wide demethylation in vivo (early embryo and PGCs) and in culture (naive ESCs), as do the oxidation and base excision repair pathways. Approximate levels of 5-methylcytosine (5mC) and 5-hydroxymethylcytosine (5hmC) are represented by blue and red lines, respectively. Dashed blue lines indicate the expected level of 5mC if demethylation was caused solely by complete inactivation of maintenance methylation.

Demethylation in PGCs occurs in two phases (Figure 2), the first during their migration to the gonad anlagen, beginning at around E8.0 (Seki et al., 2005). Careful analysis of migratory PGC demethylation over consecutive cell divisions and hairpin bisulfite analysis has revealed that this process mainly occurs by inactivation of the maintenance methylation system (Kagiwada et al., 2013; Kobayashi et al., 2013; Seisenberger et al., 2012; Ohno et al., 2013). The underlying mechanism presumably involves transcriptional downregulation of *Uhrf1* and exclusion of UHRF1 protein from the nucleus (Kagiwada et al., 2013; Magnúsdóttir et al., 2013; Seisenberger et al., 2012), as well as loss of H3K9me2, which in other systems causes DNMT1 to be mistargeted at replication foci (Estève et al., 2006; Liu et al., 2013). As such, relatively indiscriminate passive demethylation reduces global methylation levels to 30% by the time PGCs first arrive in the gonad anlagen. In addition, downregulation of the de novo methyltransferases DNMT3A, -3B, and -3L (Kurimoto et al., 2008) precludes restoration of methylation patterns following replication, contributing to rapid loss during the first wave of demethylation. Despite this widespread reduction, specific sequences remain methylated until the second wave of demethylation occurs around E11.5, including differentially methylated regions (DMRs) in imprinted loci, CGIs on the X chromosome, and germline-specific genes. Demethylation of these sequences appears to require the TET1 and TET2 proteins, which oxidize 5-methylcytosine (5mC) to 5-hydroxymethylcytosine (5hmC) (Hackett et al., 2013b; Okashita et al., 2014; Vincent et al., 2013; Yamaguchi et al., 2013a). How the TET enzymes are targeted and the ultimate fate of hydroxymethylated DNA is the subject of debate—5hmC may be passively lost by replication (currently there is no known mechanism to maintain 5hmC), or it could be subjected to further oxidation to 5-formyl- and 5-carboxyl-cytosine (5fC and 5caC, respectively), which can be actively removed by decarboxylation or base excision repair (Branco et al., 2012). Interestingly, base excision repair (BER) is also implicated in PGC demethylation (Hajkova et al., 2010), and deficiency of the deaminase AID results in 2- to 3-fold-higher levels of methylation in PGCs at E13.5 (Popp et al., 2010). However, the time and substrate of action, as well as the genomic targets of AID, are unknown. The glycosylase TDG, which can excise 5fC and 5caC as well as deaminated 5mC, is not expressed during the second window of demethylation (Kagiwada et al., 2013). Thus, while the BER pathway has been implicated in PGC demethylation, it is not clear precisely which components are involved and to what extent. Elucidating the combinatorial action of various factors during the demethylation process will need detailed studies analyzing where these factors are targeted in the genome and which downstream pathways resolve specific base modifications.

Despite the magnitude of PGC reprogramming, there are some sequences in the genome that are resistant to demethylation. These recalcitrant sequences include members of the intracisternal A particle (IAP) retrotransposon family and adjacent CGIs, as well as approximately 250 CGIs in the genome, which are variably erased (VECs) (Guibert et al., 2012; Hackett et al., 2013b; Seisenberger et al., 2012). This variable erasure may be significant because it provides a potential mechanism for transmission of epigenetic traits acquired in one generation to the next, including metabolic phenotypes recently observed in animal models (Ferguson-Smith and Patti, 2011). The large-scale erasure of epigenetic marks in the germline may explain why transgenerational epigenetic inheritance in mammals is rare. Indeed, it is more common in plants that do not undergo genome-wide epigenetic reprogramming in germ cells (Bond and Baulcombe, 2014).

#### Early Embryo

Another major wave of demethylation in the mammalian germline occurs in the early embryo, and while the magnitude of methylation loss might not be as large as in PGCs, it is perhaps more dramatic due to the precipitous loss of DNA methylation specifically in the male pronucleus (Kobayashi et al., 2012; Reik et al., 2001; Smith et al., 2012; Wang et al., 2014). Here, highly methylated sperm-derived DNA is rapidly and actively demethylated before and during the first S phase in the zygote, making it a definitive example of large-scale active demethylation (Figure 2). A number of factors have been implicated in this rapid demethylation, most prominently hydroxylation by TET3. Unlike TET1 and TET2, TET3 is highly expressed in the zygote (Gu et al., 2011; Iqbal et al., 2011; Wossidlo et al., 2011) and is required for most of the 5hmC that appears in the paternal genome upon fertilization, and for a proportion of its demethylation. Until recently, it was thought that only the paternal genome undergoes active demethylation in the early embryo; however, the discovery of considerable amounts of 5hmC and 5fC in both pronuclei suggests that it may also affect the maternal genome to some extent (Wang et al., 2014). As TDG is apparently not involved in removing these modifications, it is thought that they are lost during DNA replication (Inoue et al., 2011; Santos et al., 2013). Recent work has shown that TET3-mediated hydroxylation and demethylation is confined to S phase chromatin, while surprisingly the bulk of the demethylation occurs prior to DNA synthesis (Santos et al., 2013) (Figure 2, stages 1 and 2). AID was also shown to be required for paternal demethylation, and initial deamination may be resolved by the UNG2 glycosylase rather than TDG, opening up the intriguing possibility that cytosine deamination and long-patch BER are involved in demethylation (Santos et al., 2013). Unravelling the targets of these active demethylation pathways in the zygote and to what extent they synergize or operate in parallel will require detailed genome-wide profiling coupled with disruption of specific pathways.

As in PGCs, inactivation of the maintenance methyltransferase and de novo methylation system is thought to play a role in demethylation in preimplantation embryos following the zygote stage (Carlson et al., 1992; Hirasawa and Sasaki, 2009; Oda et al., 2013). UHRF1 protein is highly abundant in the zygote but is excluded from the nucleus together with DNMT1, resulting in passive demethylation of the maternal (and paternal) genome in the early embryo such that by E3.5 methylation in the blastocyst reaches a global methylation level of ∼25% (Figure 1) (Kobayashi et al., 2012; Smallwood et al., 2011). From basal levels in the early blastocyst, methylation slightly increases in the ICM (E3.5) and the placenta (Hon et al., 2013; Smith et al., 2012). As in migratory PGCs, some regions such as IAPs, and DMRs in imprinted genes, are protected from both active and passive demethylation in the early embryo. The mechanism by which DMRs are protected is thought to involve binding by proteins such as STELLA, ZFP57, and KAP1 and recruitment of the low level of nuclear DNMTs and UHRF1 (Li et al., 2008b; Nakamura et al., 2007, 2012; Quenneville et al., 2011).

An outstanding question in the field is the extent to which DNA demethylation contributes to the activation of the early embryo transcriptional network and acquisition of totipotency (Ishiuchi and Torres-Padilla, 2013). A large number of retrotransposon sequences are expressed in the early embryo (Evsikov et al., 2004; Kigami et al., 2003; Peaston et al., 2004). Expression of some of these repetitive sequences may be required for zygotic genome activation—inhibition of both endogenous retroviral elements (ERVs) and LINE1 elements can impair developmental competence of embryos (Beraldi et al., 2006; Kigami et al., 2003). Moreover, around 300 early embryo genes are activated by the LTRs of the MERV-L class of endogenous retroviruses through the production of chimeric transcripts (Kigami et al., 2003; Macfarlan et al., 2012), directly implicating activation of ERVs and establishment of the early embryo transcriptional network. Along with the rest of the genome, these elements are subjected to rapid DNA demethylation in the early embryo that coincides with their activation (Smith et al., 2012). Recently, a rare population of ESCs that express markers of the early embryo was identified, and as these cells can contribute to the extraembryonic lineages, they are considered to have totipotent features (Macfarlan et al., 2012; Morgani et al., 2013). Relative to other ESCs, this subpopulation has reduced methylation at MERV-L repetitive sequences, as in the early embryo. Despite this, complete removal of DNA methylation from mouse ESCs apparently does not stimulate expression of ERV elements or the transcriptional network of the early embryo (Karimi et al., 2011). Thus, the extent to which the totipotent state is dependent upon DNA demethylation, either in the context of ERVs or the transcriptional network of the early embryo in general, is currently unclear.

#### Naive ESCs

While the link between pluripotent cell types and genome-wide methylation is apparent in vivo (as witnessed by strikingly similar methylation levels of E9.5 PGCs and ICM), it was initially puzzling that ESCs conventionally grown in serum containing media exhibited high CpG methylation levels (∼80%) characteristic of somatic cells, in contrast to the hypomethylated ICM from which they are derived (Figure 1). This paradox was recently resolved; by blocking the prodifferentiation signal that serum-grown ESCs receive (by inhibiting ERK1/2 and GSK3β signaling with two small molecule inhibitors [Ying et al., 2008]), genome-wide demethylation was induced to the same extent as in preimplantation embryos (∼30%) (Ficz et al., 2013; Habibi et al., 2013). In addition to achieving striking demethylation, use of these two inhibitors (commonly known as “2i”) enabled derivation of bona fide naive ESCs from previously recalcitrant mouse strains (Kiyonari et al., 2010; Nichols et al., 2009), rats (Buehr et al., 2008; Li et al., 2008a), and even humans (Chan et al., 2013; Gafni et al., 2013). Accordingly, 2i ESCs are now considered the best cell culture representation of in vivo pluripotent stem cells.

In contrast to serum-grown ESCs, naive ESCs grown in 2i have low levels of DNMT3A/B and their targeting factor DNMT3L, suggesting a mechanistic explanation for their low methylation levels (Ficz et al., 2013; Habibi et al., 2013; Leitch et al., 2013). While knockdown of the de novo methyltransferases in serum-cultured ESCs does not immediately affect many regions thus far analyzed, LINE1 elements undergo demethylation with similar kinetics to 2i treatment following DNMT3A/B ablation (Ficz et al., 2013). These elements (and some single-copy loci) were previously shown to require continuous de novo methylation in order to maintain high levels of methylation in serum-grown ESCs (Arand et al., 2012), indicating that DNA methylation maintenance is inefficient at these loci. Thus, DNMT3A/B repression in 2i is sufficient to cause demethylation of certain genomic regions in these cells.

Demethylation in 2i occurs with similar kinetics to that of PGCs and early embryos (Figure 2), but surprisingly without global alteration of UHRF1 and DNMT1 expression (Ficz et al., 2013; Habibi et al., 2013). TET2 is upregulated by 2i treatment and hydroxylation is induced. TET1 is targeted preferentially to CGIs while TET2 is enriched at gene bodies (Huang et al., 2014), where more substantial demethylation is observed in 2i. The extent of demethylation can be enhanced by the addition of Vitamin C to 2i culture medium, which is a cofactor for activity of the TET enzymes (Blaschke et al., 2013). Interestingly, this demethylation is focused on specific sequences including germline-specific genes. Hence a significant mode of demethylation may be erosion of methylation patterns by hydroxylation and failure to repair this erosion by de novo methylation after replication.

The similarity of demethylation mechanisms between migratory PGCs, preimplantation embryos, and 2i ESCs extends to resistance to methylation erasure of IAP retrotransposons and DMRs in imprinted genes. However, with prolonged culture of ESCs in 2i, some erosion of DMR methylation does occur (Ficz et al., 2013; Hackett et al., 2013a), perhaps in part because of the consistently elevated levels of PRDM14. In that respect, prolonged 2i treatment may mimic, to some extent, the transition of migratory to gonadal PGCs.

In summary, genome-wide demethylation is achieved in naive ESCs by a combination of disabling the de novo methylation machinery and increased hydroxylation, which may occur in the context of a partially impaired maintenance methylation system (Figure 2). Further study is required to elucidate the exact contribution of these different mechanisms at distinct genomic sites and how this relates to what is known about the removal of epigenetic memory in in vivo systems.

### Reciprocal Links between Loss of Epigenetic Memory and Pluripotency

Lack of epigenetic memory is a common characteristic of pluripotent cell types and their precursors, including PGCs, ESCs, and induced pluripotent stem cells (iPSCs). This is thought to ensure that future differentiation decisions are not affected by events in the past. In support of this idea, inefficient methylation erasure at imprinted loci has been associated with a spectrum of developmental abnormalities in the progeny of *Tet1*-deleted male mice (Yamaguchi et al., 2013b). Insights into the mechanistic links between reprogramming and pluripotency are now beginning to emerge.

All experimental reprogramming techniques, including somatic cell nuclear transfer (SCNT), cell fusion, and iPSC reprogramming, involve demethylation of the genome that appears to be crucial for successfully achieving pluripotency (Apostolou and Hochedlinger, 2013; Pasque et al., 2011; Theunissen and Jaenisch, 2014 this issue of *Cell Stem Cell*). Furthermore, ESCs that lack all three methyltransferases (and are devoid of virtually all methylation) are highly resistant to differentiation, spontaneously reverting to pluripotency (Schmidt et al., 2012). Full expression of the pluripotency network is observed in both migratory PGCs and ICM cells, showing that a genomic methylation level of 30% or less is characteristically associated with the naive pluripotent state (Figure 1). This appears to be true for naive ESCs derived from mice (Ficz et al., 2013) and may be useful as a diagnostic marker for naive cells from other species (Chan et al., 2013; Gafni et al., 2013). Expression of key pluripotency factors is associated with demethylation of these loci in PGCs, in early embryos, and during experimental reprogramming (Apostolou and Hochedlinger, 2013; Hochedlinger and Plath, 2009). However, global demethylation in PGCs is not associated with promiscuous transcription (Seisenberger et al., 2012), nor is promoter demethylation in 2i associated with transcriptional activation of demethylated genes (Ficz et al., 2013; Habibi et al., 2013). Thus, while global DNA demethylation is required for activation of the pluripotency network, pluripotent cells exhibit an uncoupling of DNA methylation and transcriptional regulation. Interestingly, expression of the pluripotency network declines in gonadal PGCs (E13.5), which show further demethylation to less than 10% (Seisenberger et al., 2012). Perhaps extreme demethylation is detrimental since it may lead to genome instability especially during cell division (methylation of major satellites, for example, appears to be needed for proper chromosome segregation) (Smith and Meissner, 2013; Xu et al., 1999).

Just as DNA methylation levels potentially influence the expression of the pluripotency network, the pluripotency network can direct the machinery of epigenetic reprogramming. TET1 and TET2 are directly linked to the pluripotency network by physical interactions with NANOG (Costa et al., 2013) and PRDM14 (Okashita et al., 2014). TET1 and NANOG have overlapping patterns of DNA binding, and NANOG is required to recruit TET1 to a subset of these common loci (Costa et al., 2013). Furthermore, NANOG and TET1 act synergistically during the reprogramming of neural stem cells to iPSCs (Costa et al., 2013), and TET1 can replace OCT4 in conventional fibroblast reprogramming (Gao et al., 2013). Similarly, PRDM14 recruits TET1/2 to target loci and TET1/2 enhance PRDM14-induced DNA demethylation in ESCs (Okashita et al., 2014). DNMT3A/B/L are negatively regulated by PRDM14 (Hackett et al., 2013a; Nakaki et al., 2013; Okashita et al., 2014; Yamaji et al., 2013), in part via binding of PRDM14 to an upstream enhancer of *Dnmt3b* (Ficz et al., 2013). NANOG also binds this locus and may have similar effects on *Dnmt3b* expression. As well as the de novo methyltransferases, UHRF1 appears to be suppressed by PRDM14 in PGCs (Grabole et al., 2013; Magnúsdóttir et al., 2013). These molecular interactions conspire to ensure that DNA methylation is maintained at low levels in pluripotent cells.

In summary, the pluripotent ground state is maintained by direct interactions between the transcriptional network and the DNA methylation machinery. Cross-regulatory mechanisms ensure that robust expression of pluripotency factors is accompanied by stable hypomethylation of the genome, yielding a pluripotent ground state with little epigenetic memory.

### Creating Epigenetic Diversity at the Exit from Pluripotency

As cells exit pluripotency and begin to differentiate, new epigenetic memories are formed to define cellular identity and restrict lineage choices. The exit from pluripotency is characterized by a steep decline in TET1/2 levels and an increase in DNMT3A/B enzymes together with re-engagement of DNMT1 at replication foci (Oda et al., 2013). During this transition, cells appear to pass through an intermediate state of epigenetic priming that is characterized by high levels of both de novo methyltransferases and TET enzymes. For example, cells expressing both DNMT3B and TET1 are present in the ICM of late blastocysts (Ficz et al., 2013), and the E6.5 epiblast displays high expression of DNMT3A/B and of TET1 (Seisenberger et al., 2012). Interestingly, serum ESCs are similar to the E6.5 epiblast in the pattern and extent of DNA methylation (Ficz et al., 2013; Seisenberger et al., 2012) (Figure 1), and ESCs grown in serum conditions also express high levels of DNMT3A/B/L and of TET1/2, making them a useful model for the relatively transient primed cell population in vivo. These cells also display remarkable methylome plasticity that is highly sensitive to growth conditions. For example, the methylation status of CGIs even varies with cell passage number (Booth et al., 2012). We speculate that rapid turnover of DNA methylation may generate epigenetic heterogeneity in primed ESCs and in the epiblast and that this heterogeneity may contribute to cell fate decisions by allowing diversification prior to lineage commitment. Consistent with this hypothesis, the number of low-methylated regions (LMRs, an indirect indicator of heterogeneous methylation across a cell population) decreases as mouse ESCs differentiate to neural progenitor cells (Stadler et al., 2011). However, a decrease in the number of LMRs was not observed in a similar study of human ESC differentiation (Xie et al., 2013). Further studies utilizing single-cell approaches are necessary to resolve these discrepancies by directly analyzing cell-to-cell variation in DNA methylation.

Heterogeneity at the level of transcription has already been linked to the propensity of serum ESCs to differentiate. Reporter cell lines have shown that key pluripotency factors such as NANOG, STELLA, and REX1 are heterogeneously and dynamically expressed in these cells (Chambers et al., 2007; Hayashi et al., 2008; Toyooka et al., 2008). For example, while NANOG-low and -high cells are interchangeable, the NANOG-low population has an increased propensity to differentiate (Chambers et al., 2007) and elevated expression of differentiation markers (Kalmar et al., 2009; Singh et al., 2007). More recent studies have employed computational approaches, single-cell analyses, and live-cell imaging to demonstrate that serum ESCs are a metastable population and that cells switch between transcriptional states in a stochastic manner (Abranches et al., 2013; Chia and Ng, 2012; Kalmar et al., 2009; MacArthur et al., 2012; Mallanna and Rizzino, 2012).

Emerging evidence indicates that epigenetic mechanisms may contribute to this metastable state. One study has found that STELLA-low and -high cells have different chromatin modifications at the *Stella* promoter, and a slight increase in DNA methylation was observed in a subset of STELLA-low cells (Hayashi et al., 2008). We have also reported differences in 5mC and 5hmC between NANOG-low and -high cells, with increased expression of TET1/2 being associated with increased 5hmC in NANOG-high cells (Ficz et al., 2013). In line with these findings, it has recently been shown that transcription patterns between daughter cells differ depending on the methylation state. The hypomethylated state of ESCs grown in 2i is associated with greater transcriptional similarity between daughter cells than for ESCs grown in serum. Furthermore, cells that are deficient for the methyltransferases (TKO cells) have daughters with highly similar transcriptional patterns despite being in serum culture conditions (Jasnos et al., 2013). Generally, DNA methylation is thought to act downstream of transcriptional changes during cell fate decisions, but this fascinating result suggests that DNA methylation heterogeneity may also be able to generate transcriptional diversity in certain contexts. In support of this idea, a study of DNMT3A null hematopoietic stem cells (HSCs) has suggested that DNA methylation dynamics can direct transcriptional changes and lineage choice in response to differentiation stimuli (Challen et al., 2012). Ultimately, transcriptional and epigenetic heterogeneity are likely to be tightly linked and cross-regulatory, such that stochastic differences at either level can generate cell diversity.

**Figure 3:**
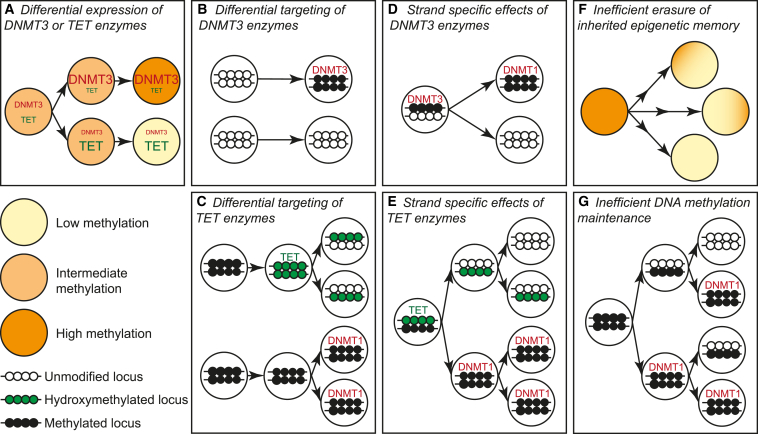
Potential Sources of DNA Methylation Heterogeneity (A) Differential expression of TET or DNMT3 enzymes between cells would lead to global changes in DNA methylation levels. (B and C) Differential recruitment of DNMT3 (B) or TET (C) enzymes at certain loci could generate cells with distinct patterns of DNA methylation. In the case of TET (C) hemi-5hmC would be an intermediate to loss of DNA methylation since DNMT1 does not maintain this mark. (D and E) Strand-specific effects of DNMT3 (D) or TET (E) enzymes could also produce daughter cells with distinct methylation patterns. In each case, hemimodified DNA would be a transitional state. (F) Erasure of inherited methylation patterns (e.g., removal of oocyte derived methylation in the ICM) could also be inefficient and stochastic, generating sister cells with distinct patterns of inherited DNA methylation. (G) Inefficient maintenance of DNA methylation could also produce DNA methylation heterogeneity via hemimethylated intermediaries.

**Figure 4:**
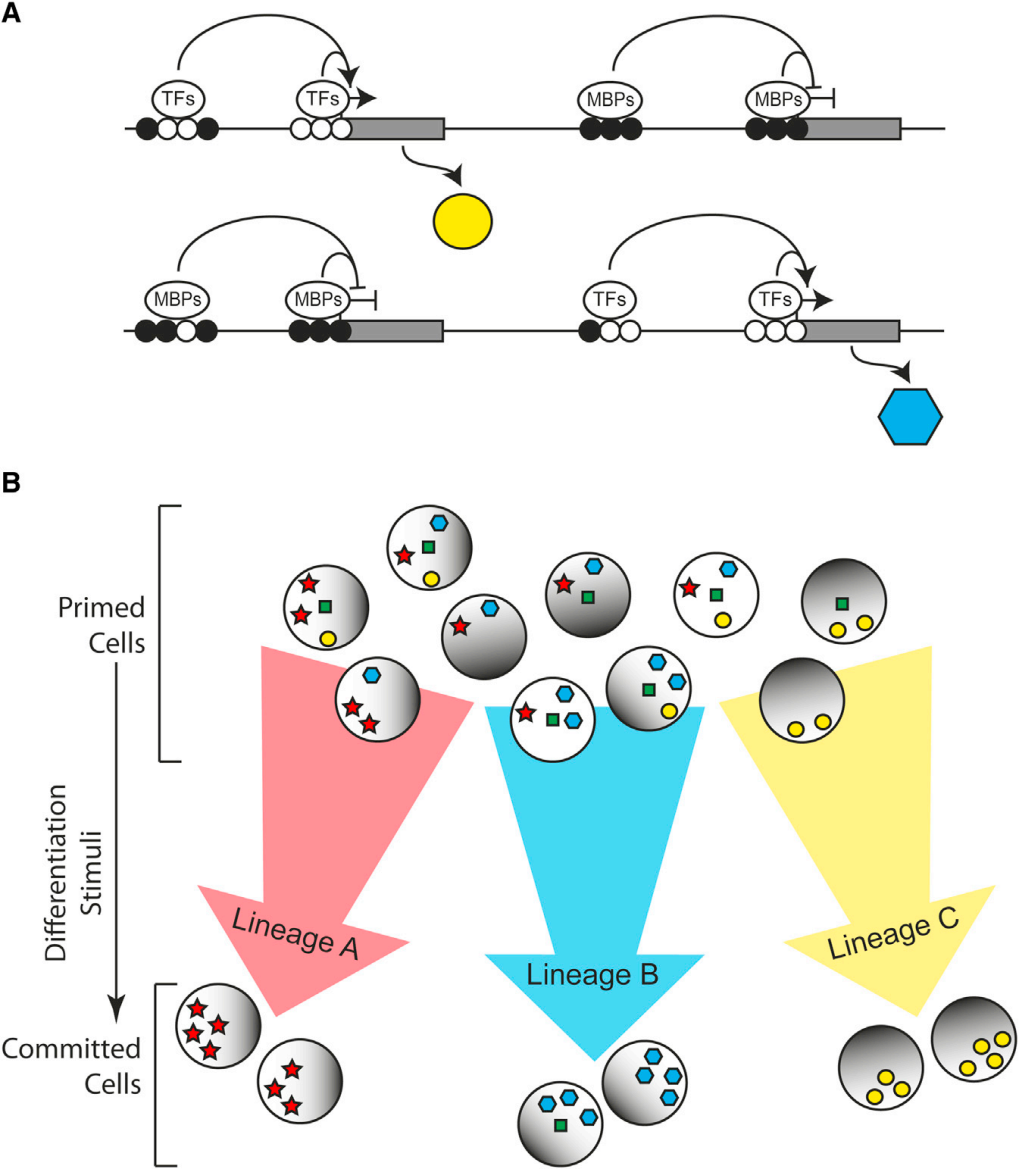
Consequences of DNA Methylation Heterogeneity for Cell Fate Decisions (A) Heterogeneous methylation at regulatory elements (e.g., enhancers and promoters) may affect the binding of transcription factors (TFs) and methyl-binding proteins (MBPs, e.g., MeCP2 and MBD1), that can in turn activate, or repress, gene expression. Black and white circles represent methylated and unmethylated sites, respectively. (B) The result is a pool of diverse cells at the exit from pluripotency in which heterogeneous patterns of methylation (black shading) underlies heterogeneous transcriptional programmes (colored shapes). This cell diversity may predispose cells toward different lineage choices upon receipt of differentiation stimuli.

DNA methylation heterogeneity may be generated by several mechanisms (Figure 3). Stochastic fluctuations in the expression of TET or DNMT3 enzymes could generate differences in DNA methylation between sister cells. Differential targeting of these enzymes could also produce cells with variable epigenetic marks at the same sequence. Alternatively, strand-specific effects of DNMT3 or TET enzymes could introduce asymmetric DNA modifications that would yield daughter cells with distinct methylation patterns. Differences in the efficiency of methylation maintenance may also lead to DNA methylation heterogeneity within a cell population, and incomplete erasure of inherited methylation patterns (e.g., oocyte-derived methylated regions) might be an additional source of epigenetic heterogeneity. The precise patterns and kinetics of such events across cell populations will depend on the balance between expression of DNMT3 and TET enzymes, as these genes are themselves heterogeneously expressed in ESCs (Ficz et al., 2013). Rapid turnover of DNA methylation through 5hmC is also likely to contribute to epigenetic heterogeneity as CGIs with variable methylation in ESCs are also enriched for 5hmC (Booth et al., 2012). Loss of DNA methylation through 5hmC will occur via hemi-methylated intermediates since there is no known mechanism for 5hmC maintenance (Figure 3). Consistently, 5hmC has been linked to hemi-methylation at repetitive elements, with LINE1 elements having increased 5hmC, increased hemi-methylation, and increased hemi-5hmC relative to IAPs (Arand et al., 2012; Ficz et al., 2013). LMRs are also enriched for 5hmC and are bound by TET1 (Stadler et al., 2011). The presence of characteristic histone modifications and transcription factor binding at LMRs predicts that these loci are distal regulatory elements. Thus, DNA methylation turnover through 5hmC and hemi-methylation may generate heterogeneous methylation at enhancers and other regulatory elements. This could modulate their transcriptional output (Figure 4), since many transcription factors display methylation-sensitive sequence binding with the majority preferring an unmethylated substrate (Hu et al., 2013; Iurlaro et al., 2013; Spruijt et al., 2013). In support of this model, methylation has been shown to inhibit the activity of lineage-specific enhancers in the context of T cell differentiation (Schmidl et al., 2009).

The epigenetically primed diversity seen in ESCs may also exist in other contexts such as hematopoietic stem cells (HSCs). In a manner analogous to ESCs, HSCs display transcriptional heterogeneity corresponding to their differentiation potential (Chang et al., 2008; Copley et al., 2012). These cells can be fractionated based on the expression of the stem cell marker SCA-1; SCA-1-high and -low subpopulations are interchangeable and are predisposed to adopt the myeloid and erythroid lineages, respectively. Loci that are hypomethylated specifically in either myeloid or lymphoid cells have intermediate levels of methylation in HSCs, and a myeloid specific locus displays stochastic DNA methylation in these cells (Hodges et al., 2011). These findings suggest that DNA methylation heterogeneity in HSCs underscores transcriptional heterogeneity and precedes lineage commitment.

In general terms, creating heterogeneity of gene expression at critical times in development is expected to help with symmetry breaking during cell fate decisions. By permitting gene expression and epigenetic heterogeneity, primed cells are able to diversify prior to lineage commitment. This diversification could allow cells to respond differently to uniform differentiation stimuli, such that multiple lineages may be initiated from the same pool of stem cells (Figure 4B). Our model thus predicts that the transitional fine-tuned overlap between methylation and demethylation systems is critical for cell fate decisions during gastrulation. This model needs to be interrogated using emerging single-cell techniques (Macaulay and Voet, 2014), which aim to decipher the complex relationships between DNA methylation and other sources of epigenetic and transcriptional heterogeneity in the same cell.

### Conclusions

Recent studies have elucidated the synergistic mechanisms that orchestrate genome-wide epigenetic reprogramming in germ cells and early embryos. Global demethylation appears to be predominantly the result of disabling the maintenance and de novo methyltransferases, while modifications of cytosine and DNA repair may be needed for more targeted demethylation events. Global hypomethylation of the genome is inextricably connected with pluripotency through reciprocal links between the DNA methylation machinery and the pluripotency transcription factor network. This ensures that naive pluripotency is essentially devoid of epigenetic memory, so that events of the past do not influence future differentiation decisions. Epigenetic memory is re-engaged at the exit from pluripotency with robust expression of the de novo and maintenance methylation systems. We propose that differentiating cells pass through a transient epigenetically primed state characterized by high levels of TET and DNMT enzymes, and heterogeneous patterns of DNA methylation. Notably, primed ESCs (grown in serum) express high levels of methyltransferases and of TET1 and 2, and similar cells appear to exist in the ICM in vivo. Epigenetic heterogeneity in the ICM and epiblast may aid cell fate decisions by allowing diversification prior to lineage commitment.

## Author Contributions

H.J.L., T.A.H., and W.R. wrote the manuscript and prepared figures.
